# Improving Completion Rates, Accuracy and Online Upload of DNA CPR, Adults With Incapacity (AWI) and Hospital Anticipatory Care Plan (HACP) Documentation in Two Old Age Psychiatry Wards in NHS Fife

**DOI:** 10.1192/bjo.2024.646

**Published:** 2024-08-01

**Authors:** Kate Womersley, Olivia Mansfield, Emma Holland, Adeeb Abuthahir, Nasser Shah

**Affiliations:** 1 NHS Lothian, Edinburgh, United Kingdom; 2 NHS Fife, Edinburgh, United Kingdom

## Abstract

**Aims:**

To evidence accurate completion and online upload of DNACPR, Adults with Incapacity (AWI) and Hospital Anticipatory Care Plan (HACP) paperwork at the point of admission across two old age psychiatry wards at Queen Margaret's Hospital, NHS Fife.

**Methods:**

We identified which of our 36 inpatients required DNA CPR, AWI and HACP forms, compared with those who actually had this documentation in place, correctly completed, in their paper notes. When documents were present, we confirmed whether they were also uploaded to Morse (NHS Fife's psychiatry electronic notes system).

Data were collected on August 25th 2023 for cycle 1. A Multidisciplinary team meeting was held in each ward to consider strategies for improving performance, and 11 weeks were allocated for intervention design and implementation, before data collection was repeated on November 10th 2023.

**Results:**

The primary outcome was whether DNA CPR, AWI and HACP documentation were correctly in place across both wards. Completion rates of all forms improved between the two cycles, as did compliance with online upload (secondary outcome) and correct completion of all fields (secondary outcome).

Since our interventions (improving availability of forms, peer education regarding correct completion of forms, ward round prompts to review paperwork, streamlining workflow for scanning), there was a marked improvement in performance on both wards 1 and 4. For patients who were assessed to need an AWI form, form completion increased from 93.3% and 94.4% for each ward respectively, to 100% on both wards. Required fields on the form were completed in 71.4% and 76.5% for each ward respectively in August, increasing to 88.2% and 100% in November. DNA CPR forms were present for appropriate patients in 100% and 88.9% of cases on the two wards in August 2023, with 75% and 62.5% uploaded to Morse. This improved to 100% presence and 100% upload rates in November 2023. HACP forms were present in 100% and 83.3% of cases on the two wards in August, but were available online in 0% and 20% of cases respectively. This improved to 100% completion of HACP forms on both wards, with 100% and 91% respectively available online in November.

**Conclusion:**

A combination of peer education, MDT learning, readily available forms, ward round review and awareness-raising across medical, nursing and administrative staff improved rates of accurate completion and online upload of DNA CPR, AWI and HACP paperwork.

